# Knowledge, attitudes, and practices of Lebanese university students related to sexually transmitted diseases: a cross-sectional study

**DOI:** 10.3325/cmj.2023.64.213

**Published:** 2023-08

**Authors:** Vanessa Sleiman, Sahar Obeid, Hala Sacre, Pascale Salameh, Souheil Hallit, Rabih Hallit

**Affiliations:** 1 School of Medicine and Medical Sciences, Holy Spirit University of Kaslik, Jounieh, Lebanon; 2 Social and Education Sciences Department, School of Arts and Sciences, Lebanese American University, Jbeil, Lebanon; 3 INSPECT-LB, National Institute of Public Health, Clinical Epidemiology and Toxicology-Lebanon, Beirut, Lebanon; 4 School of Medicine, Lebanse American University, Byblos, Lebanon; 5 Department of Primary Care and Population Health, University of Nicosia Medical School, Nicosia, Cyprus; 6 Faculty of Pharmacy, Lebanese University, Hadath, Lebanon; 7 Research Department, Psychiatric Hospital of the Cross, Jal Eddib, Lebanon; 8 Applied Science Research Center, Applied Science Private University, Amman, Jordan; 9 Department of Infectious Disease, Bellevue Medical Center, Mansourieh, Lebanon; 10 Department of Infectious Diseases, Notre Dame des Secours University Hospital Center, Byblos, Lebanon

## Abstract

**Aim:**

To appraise Lebanese university students’ knowledge, attitudes, and practices related to sexually transmitted diseases (STDs).

**Methods:**

This cross-sectional study was conducted in March and April 2020. A total of 402 students (67.9% women) from ten universities located in different Lebanese regions were recruited through convenient sampling.

**Results:**

Pursuing a major in non-health sciences compared with a major in health sciences (adjusted odds ratio [aOR] = 0.08) was significantly associated with lower odds of having better knowledge of STDs. Older age (aOR = 1.09) was significantly associated with higher odds of having better knowledge of STDs. Having good vs poor knowledge (aOR = 3.88) was significantly associated with higher odds of having favorable attitudes toward STDs, whereas pursuing a major in non-health sciences compared with a major in health sciences (aOR = 0.28) was significantly associated with lower odds of having favorable attitudes toward STDs. Women compared with men (aOR = 2.21) had higher odds of having healthier practices related to STDs. Attitude did not mediate the association between knowledge and practice.

**Conclusion:**

Attitude did not significantly mediate the association between knowledge and practice. Therefore, educational programs and awareness campaigns should be implemented in secondary schools and among vulnerable non-medical students. Such efforts can help students identify the symptoms of STDs, seek medical attention, and make informed decisions to protect themselves.

Sexually transmitted diseases (1-5) represent a significant global public health problem, despite preventive efforts and measures undertaken to tackle them (6). One million new STIs cases occur every day (7), with 110 million annual cases reported in the USA alone (8). As of July 2018, UNAIDS observed a 47% increase in global HIV cases, with a staggering 95% increase in Eastern Europe, Central Asia, and the Middle East and North Africa (MENA) region. However, considering the conservative mentality in Arab countries, including Lebanon, when it comes to sex, sexuality, and extramarital relations, data on STDs in the MENA region and Lebanon in particular are scarce (9).

The age group 15-24 years is at higher risk of contracting STDs compared with other age groups and accounts for almost 50% of the world’s STD-prone population (10). The high risk is attributed to risky sexual behavior patterns (early initiation of sex, multiple sex partners), transmission dynamics, and treatment-seeking behavior (11), all of which could result from a lack of access to reliable sexual health resources or gaps in knowledge.

Although information about STDs is available through different media and government programs, knowledge about STDs in developing countries is relatively low (3). In an Indian study (3), 90% of the surveyed students agreed on the need to include sex education in the curriculum. Similarly, in Lebanon, young people are not sufficiently familiar with sex-related topics. They gain this type of knowledge through friends, biology classes, books, and mass media, but there are no interventions by health professionals (11).

Assessing young adults’ knowledge about STDs, disease complications, and sexual health is essential to developing preventive and treatment strategies (3). Besides knowledge about STDs, attitudes and beliefs are equally important when it comes to making decisions about risky sexual practices. For instance, as their knowledge about STIs increased, unmarried youths in Nigeria showed better attitudes toward prevention measures such as using condoms and being vaccinated against the hepatitis B virus (12). Moreover, good knowledge predicted 7.8 times higher odds of having favorable attitudes (13). According to the Theory of Planned Behavior (TPB), knowledge is a critical modifier of positive attitudes (14) since people with extensive knowledge tend to have positive attitudes toward behaviors such as screening for STIs, seeking medical help, and avoiding pornography (15). However, good knowledge about HIV/AIDS and adequate attitudes toward the disease do not always translate into safe behaviors (16,17). The practice or behavior is a complex framework that is not solely determined by high levels of knowledge and favorable attitudes but also by confounding variables (18), such as attitudes toward the behavior (ie, positive or negative outcomes of the behavior), subjective norms (ie, perceived social pressure to engage or disengage in the behavior), and perceived behavioral control (ie, perceived ability to perform the behavior). According to this model, people start engaging in certain practices or behaviors once they have gained awareness, knowledge, and the desire to do so.

Moreover, STD-related knowledge, attitudes, and practices may be affected by sociodemographic factors (19), sex (11,20), and religious and cultural taboos. Additionally, stigmatization of STDs, social inequities, paradoxical media messages, and clinical preventive and curative services availability are all involved in shaping a group’s sexual health status (4).

Italian (6), Cypriot (21), and Malaysian (22) students demonstrated insufficient knowledge of STDs, while Irish students (23) exhibited higher knowledge but found it difficult to translate it to healthy practices. In Lebanon, a multi-religious and multi-cultural Middle Eastern country, research on STDs is limited. Therefore, this study aims to generate much-needed data on overall sexual practices and behaviors among young Lebanese university students.

## Participants and methods

### Study design and sample

This cross-sectional study was conducted in March and April 2020 during the COVID-19 lockdown imposed by the Lebanese government. The study used the snowball method to enroll a convenience sample of students from ten universities located in different Lebanese regions. A link to the Google Forms questionnaire was shared with university students, who were requested to further disseminate it among their friends, irrespective of their university/faculty. The questionnaire was accompanied by a statement guaranteeing the anonymity of the shared information. Participation was voluntary. No exclusion criteria were applied. The study was approved by the Ethics and Research Committee of the Psychiatric Hospital of the Cross (HPC-028-2020). Submitting the online questionnaire was deemed equivalent to giving written consent.

### Sample size calculation

Using the G-power software, we calculated a minimum sample of 395 participants based on an f2 = 2%, an error of 5%, a power of 80%, and nine factors to be entered in the multivariable analysis.

### Questionnaire

The questionnaire (Supplemental material(Supplementary Material)) was developed in the English language, based on previous studies (19,24,25). It consisted of 114 questions (divided into four parts) about the knowledge, attitudes, and practices related to STDs. As pilot-testing on 10 students revealed some overly scientific and possibly unclear terminology, the wording was modified.

The first section inquired about socioeconomic and demographic characteristics: gender, age, living conditions, education level, academic major, and religion. As students who take health science courses might have higher knowledge about STDs, the students were grouped into two categories based on the academic major: health sciences (eg, medicine, pharmacy, dentistry, nursing, and physical therapy) and non-health sciences. Living conditions referred to the number of persons living with the participant (alone or with others), the type of relationship with the participant (parents or friends), and the number of bedrooms in the students’ primary residence (excluding the bathrooms and kitchen). By dividing the number of occupants by the number of rooms in the house, we calculated the household crowding index. A higher household crowding index designated more crowded premises and indicated a lower economic status of the household.

In the second part of the questionnaire, multiple-choice or true/false questions were used to evaluate knowledge about STDs: definition, mode of transmission, symptoms, and complications. Correct answers were awarded 1 point, and incorrect answers 0. Above-average scores indicated good knowledge and below-average scores indicated poor knowledge.

The third questionnaire part inquired about the attitudes toward the prevention and spread of STDs, students’ reaction if their partner had an STD, and their behavior if they were infected. Participants rated their level of agreement with 30 statements. Each answer was assigned an internal code ranging from 1 (strongly disagree) to 5 (strongly agree). Higher scores indicated a more favorable attitude toward the subject, such as worrying about contracting STDs, regularly screening for STDs, adhering to treatment if infected, being faithful to one partner, and waiting until marriage to have sex.

The fourth section inquired about participants’ practices to determine whether their behavior affected the spread of STDs. Questions addressed sexual behavior, intravenous drug use, and communication with the partner about sexual history. Dichotomous items were rated 1 for a yes and 0 for a No. All the other questions consisted of several scenarios rated 0 (never), 1 (most of the time), 2 (sometimes), or 3 (always). Not applicable (N/A) was assigned a score of 3, equivalent to “always,” as abstinence is the most effective means of avoiding STDs. If abstinence is not an option, the best practices would be having a single partner with a known sexual history, using condoms during any sexual activity, and abstaining from sex when under the influence of alcohol or drugs. Secondary prevention consists of honesty and transparency between partners and seeking treatment for both partners if one is diagnosed with an STD.

### Statistical analysis

Since all questions in the online form were compulsory, there were no missing data. First, three exploratory factor analyses (EFA) were conducted on the knowledge/attitude/practice items with the principal component analysis technique and the Varimax extraction method (since items were not highly correlated). The models’ adequacy was confirmed with the Kaiser-Meyer-Olkin measure of sampling adequacy and Bartlett’s test of sphericity. Only the factors with an Eigenvalue higher than one were retained. The reliability of the three scales was assessed with Cronbach’s alpha or Kruder-Richardson-20 (KR20).

The results of an EFA for the factors remaining in each scale are summarized in Supplementary Table 1(Supplementary Table 1). A correlation matrix was conducted to check if the total scale score was associated with all subscales scores, which was the case for the knowledge and practice scores. Consequently, the items remaining in the EFA were summed up to form the total scores for these two variables. For the attitude scale, Factor 5 (avoidant attitude toward risky sexual practices) was not significantly associated with the total attitude score; therefore, items related to this factor were not included in the total attitude score. The remaining items were summed up to form the total attitude score (Supplementary Table 2(Supplementary Table 2), Supplementary Table 3(Supplementary Table 3), and Supplementary Table 4(Supplementary Table 4)). The KR20 value of the knowledge score was 0.82, whereas the Cronbach’s alpha values for the attitude and practice scores were 0.84 and 0.72, respectively.

The *p* values of the Kolmogorov-Smirnov test applied to the knowledge/attitude/practice scores, and their LOG transformation, were significant (*P* < 0.001 for all), confirming the non-normal distribution of our sample. Therefore, the three scores were dichotomized (low/high) according to the 75th percentile (75% correct responses) (26). χ^2^ or Fisher exact tests were used to assess the differences between categorical variables, whereas a *t* test was used to assess the differences between continuous variables. Cramer’s V value was added to determine the effect size; the values of 0.05, 0.1, 0.15, and 0.25 indicated weak, moderate, strong, and very strong effect sizes, respectively. Three logistic regressions were conducted, including each score as a dependent variable and considering all variables that showed a *P* < 0.25 in the bivariate analysis as independent variables (27). There was no multicollinearity. Finally, a path analysis was conducted with SPSS AMOS v. 29 (IBM Corp., Armonk, NY, USA) to test the indirect effect of attitude on the association between knowledge and practice. *P* < 0.05 was considered statistically significant. Statistical analysis was performed with SPSS, version 25 (IBM Corp.).

## RESULTS

### Sociodemographic characteristics

The final sample consisted of 402 students (67.9% women). The mean age was 23.08 ± 3.25 years. The majority of participants (88.8%) lived with their families, 314 (78.1%) were Christians, and 56% majored in health science. Table 1 summarizes other sociodemographic characteristics. The mean knowledge score was 32.42 ± 9.75 (min = 6; max = 52), the mean attitude score was 82.65 ± 7.80 (min = 56; max = 98), and the mean practices score was 73.42 ± 10.73 (min = 40; max = 107).

**Table 1:** Sociodemographic characteristics of the participants

Variable	N (%)
**Gender**	
male	129 (32.1)
female	273 (67.9)
**Living status**	
alone	29 (7.2)
with the family	357 (88.8)
with a friend	16 (4.0)
**Religion**	
Christian	314 (78.1)
Druze	6 (1.5)
Muslim	70 (17.4)
other	12 (3.0)
**Academic major**	
health science	225 (56.0)
non-health science	177 (44.0)
	**Mean ± standard deviation**
**Age (in years)**	23.08 ± 3.25
**House crowding index**	0.93 ± 0.44

### Bivariate analysis

The results of the bivariate analysis are presented in Table 2. Significantly more participants with health science majors had better knowledge about STDs than those with non-health science majors. Significantly more participants with health science majors and those with high knowledge scores had favorable attitudes toward STDs. Significantly more women and participants with better knowledge engaged in healthier/safer practices related to STDs.

**Table 2:** Bivariate analysis of factors associated with knowledge, attitude, and practice*

Variable	Knowledge				Attitude				Practice			
	low	high	*p*	effect size	low	high	*p*	effect size	low	high	*p*	effect size
**Gender**			0.806	0.016			0.714	0.021			**0.005**	0.142
male	95 (73.6)	34 (26.4)			98 (76.0)	31 (24.0)			107 (82.9)	22 (17.1)		
female	205 (75.1)	68 (24.9)			202 (74.0)	71 (26.0)			190 (69.6)	83 (30.4)		
**Living status**			0.200	0.090			0.695	0.044			0.473	0.062
alone	21 (72.4)	8 (27.6)			23 (79.3)	6 (20.7)			19 (65.5)	10 (34.5)		
with the family	264 (73.9)	93 (26.1)			264 (73.9)	93 (26.1)			265 (74.2)	92 (25.8)		
with a friend	15 (93.8)	1 (6.3)			13 (81.3)	3 (18.8)			13 (81.3)	3 (18.8)		
**Religion**			0.581	0.032			0.096	0.087			0.130	0.082
Christian	232 (73.9)	82 (26.1)			228 (72.6)	86 (27.4)			226 (72.0)	88 (28.0)		
Muslim	68 (77.3)	20 (22.7)			72 (81.8)	16 (18.2)			71 (80.7)	17 (19.3)		
**Major of studies**			**<0.001**	0.402			**<0.001**	0.321			0.820	0.014
health science	133 (59.1)	92 (40.9)			140 (62.2)	85 (37.8)			165 (73.3)	60 (26.7)		
non-health science	167 (94.4)	10 (5.6)			160 (90.4)	17 (9.6)			132 (74.6)	45 (25.4)		
**Knowledge**							**<0.001**	0.383			**0.037**	0.109
low					253 (84.3)	47 (15.7)			230 (76.7)	70 (23.3)		
high					47 (46.1)	55 (53.9)			67 (65.7)	35 (34.3)		
**Attitude**											0.067	0.096
low									229 (76.3)	71 (23.7)		
high									68 (66.7)	34 (33.3)		
**Age**	22.93 ± 3.44	23.54 ± 2.55	0.102	0.201	22.99 ± 3.44	23.37 ± 2.57	0.233	0.125	22.97 ± 3.30	23.41 ± 3.09	0.233	0.137
**Household crowding index**	0.95 ± 0.44	0.87 ± 0 .43	0.153	0.178	0.93 ± 0.46	0.92 ± 0 .41	0.885	0.022	0.95 ± 0 .45	0.86 ± 0.43	0.060	0.204

*Data are presented as n (%) if not otherwise indicated.

### Multivariable analysis

The first logistic regression, involving the dichotomized knowledge score as the dependent variable, showed that pursuing a major in non-health sciences compared with a major in health sciences (adjusted odds ratio [aOR] = 0.08) was significantly associated with lower odds of having better knowledge about STDs. However, older age (aOR = 1.09) was significantly associated with higher odds of having better knowledge about STDs (Table 3, Model 1).

**Table 3:** Multivariable analysis of factors associated with knowledge, attitude, and practice*

	Adjusted odds ratio	*p*	95% confidence interval
Model 1: Logistic regression including the knowledge score (high vs low*) as the dependent variable (Nagelkerke R^2^ = 0.272)			
Living status		0.481	
alone	1		
with the family	0.76	0.596	0.28; 2.08
with a friend	0.24	0.226	0.02; 2.44
Academic major (non-health vs health science*)	0.08	**<0.001**	0.04; 0.17
Age	1.09	**0.028**	1.01; 1.19
Household crowding index	0.83	0.542	0.45; 1.53
Model 2: Logistic regression including the attitude score (high vs low*) as the dependent variable (Nagelkerke R^2^ = 0.252)			
Academic major (non-health vs health science*)	0.28	**<0.001**	0.15; 0.51
Religion (Muslims vs Christians*)	0.58	0.095	0.30; 1.10
Knowledge (high vs low*)	3.88	**<0.001**	2.25; 6.67
Age	1.03	0.493	0.95; 1.12
Model 3: Logistic regression taking the practice score (high vs low*) as the dependent variable (Nagelkerke R^2^ = 0.073)			
Gender (female vs male*)	2.21	0.004	1.30; 3.77
Religion (Muslims vs Christians*)	0.67	0.195	0.37; 1.23
Age	1.03	0.423	0.96; 1.10
Household crowding index	0.65	0.129	0.37; 1.13
Knowledge (high vs low*)	1.49	0.148	0.87; 2.57
Attitude (high vs low*)	1.33	0.308	0.77; 2.29

*Reference group.

The second logistic regression, including the dichotomized attitude score as the dependent variable, showed that having good vs poor knowledge (aOR = 3.88) was significantly associated with higher odds of having favorable attitudes toward STDs. Moreover, pursuing a major in non-health sciences compared with a major in health sciences (aOR = 0.28) was significantly associated with lower odds of having favorable attitudes toward STDs (Table 3, Model 2).

The third logistic regression, including the dichotomized practice score as the dependent variable, showed that being female compared with being male (aOR = 2.21) was significantly associated with higher odds of having healthier/safer practices toward STDs (Table 3, Model 3).

### Path analysis

Attitude did not mediate the association between knowledge and practice (Beta = 0.024; *P* = 0.256; 95% confidence interval -0.018-0.071). Better knowledge was significantly associated with more favorable attitudes (Beta = 0.38; *P* < 0.001), whereas attitude was not significantly associated with practice (Beta = 0.06; *P* = 0.237). Finally, knowledge was not significantly and directly associated with practice (Beta = 0.09; *P* = 0.115).

## DISCUSSION

In this study, pursuing a major in non-health sciences compared with a major in health sciences was associated with lower knowledge and less favorable attitudes toward STDs. Older age was associated with better knowledge, and better knowledge was associated with more favorable attitudes toward STDs. Finally, women had healthier/safer practices related to STDs than men.

### Knowledge

Our results showed that older age was significantly associated with higher odds of having good knowledge about STDs, contrary to some other findings (28) but consistent with others (6,24,29,30). According to some authors, older students potentially have more sexual experience and have been exposed to elementary information about STDs (24).

In line with previous findings (24,29,31), students majoring in health sciences had higher knowledge, as the curricula of these majors often include sexual education. However, a study from Saudi Arabia (30) did not find students majoring in health sciences to have greater knowledge compared with other students.

### Attitude

In this study, higher knowledge scores were associated with a more favorable attitude toward STDs. Previous studies (1,32) showed that better knowledge helped young adolescents understand the importance of various prevention methods and improve their attitudes. However, a Portuguese study (28) found that students with moderate knowledge levels had highly safe attitudes. Some hypotheses recognize that knowledge is the basis of people’s attitudes and practices (28).

Furthermore, pursuing a major in non-health sciences compared with a major in health sciences was significantly associated with lower odds of having favorable attitudes toward STDs. Previous studies showed a correlation between higher levels of knowledge, favorable attitudes, and involvement in the medical field (33). Indeed, participants enrolled in medical or health-sciences programs receive information about STDs from reliable sources as part of their curriculum, a fact likely contributing to their more favorable attitudes toward the subject. These results underscore the role of health care professionals in raising public awareness about this topic. Medical personnel could leverage their knowledge to inform the public about the risks associated with STDs and their prevention.

### Practice

Our results showed that women had healthier/safer practices related to STDs than men. Previous reports (6,32,34,35) showed that women had higher levels of concerns and more prudent practices regarding safety issues during sex. Men are also more sexually active (11), probably because preserving virginity is still an important moral issue among women, especially in Middle-Eastern countries. Thus, a more liberal attitude toward sex, more frequent among men, fosters risky sexual activities with a subsequent occurrence of unwanted pregnancies and higher vulnerability to STDs.

### Mediation analysis

Finally, our results showed that attitude did not mediate the association between knowledge and practice. Indeed, better knowledge was significantly associated with more favorable attitudes, whereas knowledge and attitude were not significantly associated with practices. These results partially agree with those of previous studies (28,34), confirming that knowledge does not predict behavioral outcomes and that the best predictor of behavioral changes is experience rather than knowledge (36).

Overall, a higher level of knowledge leads to a healthier attitude toward sexual health, which, in turn, is associated with safer practices; often, improved knowledge and attitude lead to improved practice (13). A link has been established between attitudes and knowledge about STDs (13,16,17), and having good baseline knowledge increases the likelihood of having a positive attitude. However, this study did not find a significant relationship between knowledge and attitudes toward behaviors/practices such as abstinence, using and giving recommendations about condoms, and stigmatization of people living with HIV/AIDS. For instance, in Middle-Eastern cultures like Lebanon, condoms are often associated with free premarital sex behavior, which contradicts local values and norms (37). Furthermore, giving advice or condoms to friends is uncommon in Lebanese society because it may lead to stigmatization. These findings may help stakeholders in their efforts to reduce STI transmission through frequent condom use, by focusing on prevention over stigma, social norms, or individual subjectivity issues (38). For this purpose, educational policies should incorporate sex education and change how messages are delivered to promote knowledge and positive attitudes while emphasizing the appropriate practices for STD prevention.

### Limitations

Our study has some limitations. The cross-sectional design prevents us from drawing any conclusions on the causality between knowledge about STDs and associated factors. Although the study enrolled a large sample of students from all over Lebanon, the results cannot be generalized to all Lebanese students since the majority of participants were women and medical and paramedical students. Due to the use of the snowball technique and the unknown refusal rate, a selection bias is also possible. We tried to minimize this type of bias by applying weighting adjustment to the general population. Since the questionnaire was in English, we cannot be sure that only students from Lebanon took part in the study. Moreover, information bias is possible as some questions might have been incompletely understood and some items of the scales loaded inversely on their respective factors, although their codes were reversed before the analysis. Also, as the self-report method was used, responses may not reflect the actual sexual knowledge, intentions, and practices of the participants, given the cultural and religious conservative norms prevailing in the country. The scales were not pretested for psychometric properties during piloting for comprehension. Internal consistency measures may be acceptable for all three composite measures, but this is partly due to the large number of items comprising each of the measures (the value of both Cronbach’s alpha and KR-20 can be inflated as the number of items increases). Finally, residual confounding bias is also possible since not all the factors associated with knowledge, attitudes, and practices toward STDs could be controlled for. Further longitudinal studies addressing these limitations are necessary to confirm our findings.

### Conclusion

Our findings showed that attitude did not significantly mediate the association between knowledge and practice. The results of this study highlight potential gaps in sexual health prevention programs. Given the scarcity of data and the current economic crisis, a more targeted approach is necessary to guide nationwide interventions and actions. These interventions should include updating educational programs in schools and universities, launching awareness campaigns, and allocating more funds for sexual health clinics.
