# Supplementary material for: Knowledge, attitudes, and practices of Lebanese university students related to sexually transmitted diseases: a cross-sectional study
Source: Croat Med J. 2023 Aug;64(4):213–21. doi: 10.3325/cmj.2023.64.213 (PMC10509678; doi:10.3325/cmj.2023.64.213)
Supplement: Supplementary Material [file CroatMedJ_64_s001.pdf]

❖ *SOCIO-DEMOGRAPHIC CHARACTERISTICS*

1. **Gender:** □ male □ female
2. **Age:** ….
3. **With whom do you live?** □ Alone □ family □ friends
4. **What is your Education Level?** □Illiterate □Primary □Complementary □Secondary □University
5. **Faculty Type:** □ Health Sciences □Non-Health Sciences
6. **Religion:** □ Christian □ Druze □ Muslim □Other
7. **How many people (including yourself) live in your home? …….**
8. **How many *bedrooms (including guest bedrooms, bedrooms used as offices etc) are in the house, which is your PRIMARY residence? ….***

❖ *Knowledge:*

1. **Have you ever heard of STDs?** □Yes □ No
2. **Which of the following is an STD?** □Tuberculosis □HIV/AIDS □Chlamydia □Hepatitis B □ Hepatitis C □Gonorrhea □Syphilis □Genital herpes □Trichomoniasis
3. **What are the causative organisms of STDs?** □Bacteria □Virus □Fungi □Parasites □Mosquitoes
4. **What are the routes of transmission of STDs?** □ Sexual intercourse □Blood transfusion □ Sharing injection needles □Sharing food/drinks □ Sharing clothes □Infected mother to child □ Kissing
5. **What are the symptoms of STDs?**
   □Ulcers in the genital organ □Pain while passing out urine □Swollen glands, fever and body ache □Discharge from the penis □Discharge from the vagina □Itching around the vagina □Sore throat □Painless sores on the mouth and genital area
6. **Can people with STDs have no symptoms?** □yes □no □I don't know
7. **What are the complications of STDs?** □Infertility □Cervical cancer □Body weakness □Ectopic pregnancy □Still birth

| | True | False | I don't know |
|---|---|---|---|
| 1. Use of contraceptive pills can reduce risk of STDs | | | |
| 2. Use of condoms can decrease the risk of being infected with an STD | | | |
| 3. Monogamy can reduce one's chance of infection | | | |
| 4. Alcohol intake can increase an individual's susceptibility to STDs | | | |
| 5. Intake of some drugs can increase an individual's susceptibility to STDs | | | |

| | | | |
|---|---|---|---|
| 6. Having multiple sexual partners can increase chances of being infected sexually | | | |
| 7. Abstinence is the most effective means of avoiding STDs | | | |
| 8. Genital Herpes is caused by the same virus as HIV | | | |
| 9. Frequent urinary infections can cause Chlamydia | | | |
| 10. It is easier to get HIV if a person has another Sexually transmitted disease | | | |
| 11. Human Papillomavirus (HPV) is caused by the same virus that cause HIV | | | |
| 12. Having anal sex increases a person's risk of getting Hepatitis B | | | |
| 13. Soon after infection with HIV a person develops open sores on his or her genitals (penis or vagina) | | | |
| 14. A woman who has Genital Herpes can pass the infection to her baby during childbirth | | | |
| 15. There is a cure for Chlamydia | | | |
| 16. A woman can look at her body and tell if she has Gonorrhea | | | |
| 17. The same virus causes all of the sexually transmitted diseases | | | |
| 18. HPV can cause Genital Warts | | | |
| 19. Using a natural skin (lambskin) condom can protect a person from getting HIV | | | |
| 20. HPV can lead to cancer in women | | | |
| 21. A man must have vaginal sex to get Genital Warts | | | |
| 22. Sexually transmitted disease can lead to health problems that are usually more serious for men than women | | | |
| 23. A woman can tell she has Chlamydia if she has a bad smelling odor from her vagina | | | |
| 24. If a person tests positive for HIV the test can tell how sick the person will become | | | |
| 25. There is a vaccine available to prevent a person from getting Gonorrhea | | | |
| 26. A woman can tell by the way her body feels if she has a sexually transmitted disease | | | |
| 27. A person ho has genital herpes must have open sores to give the infection to his or her sexual partner | | | |
| 28. There is a vaccine that prevents a person from getting Chlamydia | | | |
| 29. A man can tell by the way his body feels if he has Hepatitis B | | | |
| 30. If a person had Gonorrhea in the past he or she is immune (protected) from getting it again | | | |
| 31. HPV can cause HIV | | | |
| 32. A man can protect himself from getting Genital Warts by washing his genitals after sex | | | |

**From where have you received information on sexually transmitted diseases? (You can mark more than one alternative)** □ Friends□ Television□ Internet□ Hospital/Clinic□ Family

_ATTITUDE:_

| | Strongly agree | Agree | Disagree | Strongly disagree |
|---|---|---|---|---|
| 1. I feel condoms protect people against STDs | | | | |
| 2. I feel it is not necessary to use condom during anal sex | | | | |
| 3. If both partners are infected with STDs, I feel there is no need of using a condom | | | | |
| 4. I feel numerous sexual partners play no role in STDs transmission | | | | |
| 5. I feel condoms play an important role in preventing STDs | | | | |
| 6. I feel it is not necessary for academic institutions to discuss issues regarding prevention of STDs | | | | |
| 7. I feel banning of prostitution can control the spread of STDs | | | | |
| 8. I feel screening for STDs is good | | | | |
| 9. I feel screening for STDs before marriage is important | | | | |
| 10. I think watching/reading pornographic materials can contribute to risky sexual practices | | | | |
| 11. STDs are not dangerous because they can be cured | | | | |
| 12. In my opinion, I feel STDs can cause death if left untreated | | | | |
| 13. I am worried about contracting STDs | | | | |
| 14. The STD problem is something that I have not given much thought to | | | | |
| 15. Homosexual men are solely to be blamed for the spread of STDs | | | | |
| 16. If I notice symptoms of STDs, I think I should seek treatment immediately | | | | |
| 17. If I notice symptoms of STDs in my partner, I will advise him/her to seek treatment immediately | | | | |
| 18. Getting early medical care is the main key to prevent the harmful effects of STIs | | | | |
| 19. If a woman suffers from STIs she should inform her partner or spouse | | | | |

| | | | | |
|---|---|---|---|---|
| 20. In case of STIs, I take medication concurrently with my partner | | | | |
| 21. I will not go to the doctor unless I am certain I have STIs | | | | |
| 22. I do not like to talk about STIs with my relatives/ friends | | | | |
| 23. STIs cannot be treated and lasts for life | | | | |
| 24. Poverty may force women to prostitution | | | | |
| 25. I will avoid sexual contact anytime I think there is even a slight chance of getting f I had STIs | | | | |
| 26. I would avoid exposing others during treatment | | | | |
| 27. It is easy to use the STI prevention methods | | | | |
| 28. Women's examination is a shame | | | | |
| 29. I would dislike having to follow the medical steps for treating STIs | | | | |
| 30. I always follow up with treatment after cure I will have less fear of STIs by receiving information about them | | | | |

**31. If I have unprotected sexual intercourse, I am most concerned about:**

□ *Getting HIV*

□ *Getting STDs aside from HIV*

□ *Unwanted pregnancy*

❖ <u>*Practice:*</u>

1. **Do you abstain from sex?** □Yes □No □I DON'T KNOW
2. **Was a condom used the last time you had sex?** □Yes □No □I DON'T KNOW
3. **Do you have sex with only one partner for the past 12 months?** □Yes □No □I DON'T KNOW
4. **If you ever received a blood transfusion, was the blood screened?** □Yes □No □I DON'T KNOW
5. **Do you get tested for STDs annually?** □Yes □No □I DON'T KNOW
6. **Does your partner get tested for STDs annually?** □Yes □No □I DON'T KNOW
7. **Do you share injection needle with others?** □Yes □No □I DON'T KNOW
8. **Do you read pornographic materials?** □Yes □No □I DON'T KNOW
9. **Do you have sex with commercial sex workers?** □Yes □No □I DON'T KNOW

| | ALWAYS | MOST OF THE TIME | SOMETIMES | NEVER |
|---|---|---|---|---|
| 1. I insist on condom use when I have sexual intercourse. | | | | |
| 2. I use cocaine or other drugs prior to or during sexual intercourse. | | | | |
| 3. I stop foreplay long enough to put on a condom (or for my partner to put on a condom). | | | | |
| 4. I ask potential sexual partners about their sexual histories. | | | | |
| 5. I avoid direct contact with my sexual partner's semen or vaginal secretions. | | | | |
| 6. My partner and I use spermicide as well as a condom with each act of sexual intercourse. | | | | |
| 7. I have sexual intercourse with someone who injects drugs (IV drugs) into his/her veins. | | | | |
| 8. I ask my potential sexual partners about a history of bisexual/homosexual practices. | | | | |
| 9. I engage in sexual intercourse on a first date. | | | | |
| 10. I abstain from sexual intercourse when I do not know my partner's sexual history. | | | | |
| 11. I avoid sexual intercourse when I have sores or irritation in my genital area. | | | | |
| 12. If I know an encounter may lead to sexual intercourse, I carry a condom with me. | | | | |
| 13. I insist on examining my sexual partner for sores, cuts, or abrasions in the genital area. | | | | |
| 14. If I disagree with information that my partner presents on safer sex practices, I state my point of view. | | | | |
| 15. I engage in oral sex without using protective barriers such as a condom or rubber dam. | | | | |
| 16. I use rubber gloves for sexual foreplay when I have cuts or abrasions on my hands. | | | | |
| 17. If swept away in the passion of the moment, I have sexual intercourse without using a condom. | | | | |

| | | | | |
|---|---|---|---|---|
| 18. I engage in anal intercourse. | | | | |
| 19. I ask my potential sexual partners about a history of IV drug use. | | | | |
| 20. If I know an encounter may lead to sexual intercourse, I have a mental plan to practice safer sex. | | | | |
| 21. If my partner insists on sexual intercourse without a condom, I refuse to have sexual intercourse. | | | | |
| 22. I avoid direct contact with my sexual partner's blood. | | | | |
| 23. It is difficult for me to discuss sexual issues with my sexual partners. | | | | |
| 24. I initiate the topic of safer sex with my potential sexual partner. | | | | |
| 25. I have sexual intercourse with someone who I know is a bisexual or gay. | | | | |
| 26. I engage in anal intercourse without using a condom. | | | | |
| 27. I drink alcoholic beverages prior to or during sexual intercourse. | | | | |
