# Supplementary material for: Knowledge, attitudes, and practices of Lebanese university students related to sexually transmitted diseases: a cross-sectional study
Source: Croat Med J. 2023 Aug;64(4):213–21. doi: 10.3325/cmj.2023.64.213 (PMC10509678; doi:10.3325/cmj.2023.64.213)
Supplement: Supplementary Table 1 [file CroatMedJ_64_s002.pdf]

**Supplementary Table 1. Exploratory Factor Analyses using the principle component analysis of the knowledge, attitude and practice scales' items using the promax rotation.**						
	**F1**	**F2**	**F3**	**F4**	**F5**	**F6**
**Model 1: Knowledge scale items (KMO= .895; Bartlett's test of sphericity p <.001; variance explained = 58.07%)**						
Human papilloma virus (HPV) is caused by the same virus that causes HIV	.81					
HPC can cause HIV	.75					
Genital herpes is caused by the same virus as HIV	.75					
HPV can lead to cancer in women	.72					
There is a cure for chlamydia	.68					
Frequent urinary infections can cause chlamydia	.67					
There is a vaccine that prevents a person from getting chlamydia	.60					
A woman who has genital herpes can pass the infection to her baby during childbirth	.60					
Soon after infection with HIV a person develops open sores on his/her genitals	.59					
There is a vaccine available to prevent a person from getting gonorrhea	.50					
Symptoms of STD		.79				
Routes of transmission of STDs		.78				
Complications of STD		.75				
Which of the following is an STD		.56				
A woman can tell by the way her body feels if she has an STD			.73			
A woman can tell she has chlamydia if she has a bad smelling odor from her vagina			.68			
STD can lead to health problems that are usually more serious for men than women			.63			
It is easier to get HIV if a person has another STD				.77		
Having anal sex increases a person's risk of getting hepatitis B				.65		
Ever heard of STDs					.80	

Use of condoms can decrease the risk of being infected with an STD					.66	
Using a natural skin (lambskin) condom can protect a person from getting HIV						.94
F1: Knowledge about HIV Etiology, consequences and treatment; F2: Knowledge about symptoms and complications of STDs; F3:: Knowledge about STD and gender; F4: Knowledge about risk factors for HIV and Hepatitis B ; F5: Prior knowledge about STDs; F6: Knowledge about HIV preventive measures						
**Model 2: Attitude scale items (KMO= .869; Bartlett's test of sphericity p <.001; variance explained = 60.14%)**						
Women's examination is a shame	.76					
I would dislike having to follow the medical steps for treating STDs	.75					
I feel it is not necessary for academic institutions to discuss issues regarding the prevention of STDs	.75					
STDs are not dangerous because they can be cured	.73					
If both partners are infected with STDs, I feel there is no need for using a condom	.71					
I feel numerous sexual partners play no role in STDs transmission	.67					
I feel it is not necessary to use condoms during anal sex	.67					
If I notice symptoms of STDs in my partner, I will advise him/her to seek treatment immediately		.89				
If a woman suffers from STDs, she should inform her partner of spouse		.85				
If I notice symptoms of STDs, I think I should seek treatment immediately		.85				
Getting early medical care is the main key to prevent the harmful effects of STDs		.79				
In case of STDs, I take medications concurrently with my partner		.54				
It is easy to use the STD prevention method			.78			
I feel condoms protect people against STDs			.66			
I feel screening for STDs is good			.65			
I would avoid exposing others during treatment			.61			
I follow up with treatment after cure			.52			
I do not like to talk about STDs with my relatives/friends				.81		
I will not go to the doctor unless I am certain I have STDs				.64		
I feel banning of prostitution can control the spread of STDs					.76	
I think watching/reading pornographic materials can contribute to risky sexual practices					.75	
I am worried about contracting an STD						.88
F1: Shame and inappropriate attitudes towards STDs' preventive measures; F2: Treatment-seeking behaviors; F3: Appropriate attitudes towards preventive measures; F4: Defensive attitudes; F5: Avoidant attitude of risky sexual practices (encountering sex workers and watching pornography); F6: Worry about contracting STD						
**Model 3: Practice scale items (KMO= .817; Bartlett's test of sphericity p <.001; variance explained = 57.31%)**						

I engage in anal intercourse without using a condom	.84					
I engage in anal intercourse	.76					
If swept away in the passion of the moment, I have sexual intercourse without using a condom	.73					
I have sexual intercourse with someone who I know is a bisexual or gay	.72					
It is difficult for me to discuss sexual issues with my sexual partners	.68					
I drink alcoholic beverages prior to or during sexual intercourse	.61					
I have sexual intercourse with someone who injects drugs into his/her veins	.55					
I engage in sexual intercourse on a first date	.53					
I use cocaine or other drugs prior to or during sexual intercourse	.52					
I engage in oral sex without using protective barriers such as a condom or rubber dam	.49					
I ask my potential sexual partners about a history of bisexual/homosexual practices		.80				
I abstain from sexual intercourse when I do not know my partner's sexual history		.76				
I ask potential sexual partners about their sexual histories		.75				
I ask my potential sexual partners about a history of intravenous drug use		.61				
If I disagree with information that my partner presents on safer sex practices, I state my point of view.		.42				
I insist on condom use when I have sexual intercourse			.72			
I stop foreplay long enough to put on a condom (or for my partner to put a condom)			.70			
If my partner insists on sexual intercourse without a condom, I refuse to have sexual intercourse			.62			
Was a condom used the last time you had sex?			.57			
I use rubber gloves for sexual foreplay when I have cuts or abrasions on my hands.				.75		
My partner and I use spermicide as well as a condom with each act of sexual intercourse.				.75		
I avoid direct contact with my sexual partner's semen or vaginal secretions.				.53		
Do you have sex with commercial sex workers?					.83	
Do you share injection needs with others?					.75	
If I know an encounter may lead to sexual intercourse, I have a mental plan to practice safer sex						.62
Have you had sex with only one partner for the past 12 months?						-.57
I insist on examining my sexual partner for sores, cuts, or abrasions in the genital area.						.51
I avoid direct contact with my sexual partner's blood.						.46
F1: Risky sexual practices; F2: Partners' communication about sexual histories; F3: Preventive measures by using condoms; F4: Safe sex practices; F5: Practices related to sex workers and needles use; F6: Mental plan to practice safer sex						
