# Supplementary material for: Knowledge, attitudes, and practices of Lebanese university students related to sexually transmitted diseases: a cross-sectional study
Source: Croat Med J. 2023 Aug;64(4):213–21. doi: 10.3325/cmj.2023.64.213 (PMC10509678; doi:10.3325/cmj.2023.64.213)
Supplement: Supplementary Table 2 [file CroatMedJ_64_s003.pdf]

**Supplementary Table 2. Correlation matrix of the knowledge total score and its subscales.**							
	**Total score**	**F1**	**F2**	**F3**	**F4**	**F5**	**F6**
Total score	1						
F1	.84***	1					
F2	.91***	.57***	1				
F3	.49***	.47***	.27***	1			
F4	.27***	.20***	.14**	.11	1		
F5	.28***	.19***	.22***	.11*	.002	1	
F6	.17**	.15**	.08	.11*	.02	.06	1

*p <.05; **p <.01; ***p <.001
