# Supplementary material for: Knowledge, attitudes, and practices of Lebanese university students related to sexually transmitted diseases: a cross-sectional study
Source: Croat Med J. 2023 Aug;64(4):213–21. doi: 10.3325/cmj.2023.64.213 (PMC10509678; doi:10.3325/cmj.2023.64.213)
Supplement: Supplementary Table 3 [file CroatMedJ_64_s004.pdf]

Supplementary Table 3. Correlation matrix of the attitude total score and its subscales.							
	**Total score**	**F1**	**F2**	**F3**	**F4**	**F5**	**F6**
Total score	1						
F1	.77***	1					
F2	.71***	.29***	1				
F3	.68***	.27***	.53***	1			
F4	.48***	.42***	.17***	.13**	1		
F5	.08	-.21***	.03	.03	-.11*	1	
F6	.13**	-.07	.09	.10*	-.09	.05	1

*p <.05; **p <.01; ***p <.001
