# Supplementary material for: Knowledge, attitudes, and practices of Lebanese university students related to sexually transmitted diseases: a cross-sectional study
Source: Croat Med J. 2023 Aug;64(4):213–21. doi: 10.3325/cmj.2023.64.213 (PMC10509678; doi:10.3325/cmj.2023.64.213)
Supplement: Supplementary Table 4 [file CroatMedJ_64_s005.pdf]

Supplementary Table 4. Correlation matrix of the practice total score and its subscales.							
	**Total score**	**F1**	**F2**	**F3**	**F4**	**F5**	**F6**
Total score	1						
F1	.46***	1					
F2	.61***	-.16--	1				
F3	.56***	.02	.28***	1			
F4	.48***	-.02	.21***	.15**	1		
F5	.19***	.10*	.16***	.11*	-.11*	1	
F6	.60***	-.19***	.42***	.52***	.29***	.04	1

*p <.05; **p <.01; ***p <.001
